# Research on PM2.5 Spatiotemporal Forecasting Model Based on LSTM Neural Network

**DOI:** 10.1155/2021/1616806

**Published:** 2021-10-19

**Authors:** Fang Zhao, Ziyi Liang, Qiyan Zhang, Dewen Seng, Xiyuan Chen

**Affiliations:** ^1^School of Computer Science and Technology, Zhejiang Shuren University, Hangzhou 310015, China; ^2^School of Computer Science and Technology, Hangzhou Dianzi University, Hangzhou 310018, China

## Abstract

Accurate monitoring of air quality can no longer meet people's needs. People hope to predict air quality in advance and make timely warnings and defenses to minimize the threat to life. This paper proposed a new air quality spatiotemporal prediction model to predict future air quality and is based on a large number of environmental data and a long short-term memory (LSTM) neural network. In order to capture the spatial and temporal characteristics of the pollutant concentration data, the data of the five sites with the highest correlation of time-series concentration of PM2.5 (particles with aerodynamic diameter ≤2.5 mm) at the experimental site were first extracted, and the weather data and other pollutant data at the same time were merged in the next step, extracting advanced spatiotemporal features through long- and short-term memory neural networks. The model presented in this paper was compared with other baseline models on the hourly PM2.5 concentration data set collected at 35 air quality monitoring sites in Beijing from January 1, 2016, to December 31, 2017. The experimental results show that the performance of the proposed model is better than other baseline models.

## 1. Introduction

In recent years, with the rapid development of society, the pressure on the environment has become more and more serious, and some serious air pollution problems have seriously threatened people's health. In the case of cardiovascular disease, exposure to PM2.5 (fine particulate matter with a particle size of less than 2.5 *μ*m) as short as several hours to several weeks can lead to an increase in mortality, which can lead to a reduction in lifespan for up to several years. Lowering the concentration of PM2.5 can effectively reduce the above risks [1]. In addition, there are more and more studies on the relationship between gas pollution and neurodegenerative diseases such as Alzheimer's disease. A 2015 study showed that prolonged exposure to PM2.5 can lead to an advance in the first outpatient time for neurodegenerative diseases, with more than 3 million premature deaths per year due to PM2.5 exposure [2–5]. Therefore, accurate prediction of its mass concentration plays a key role in atmospheric management decisions [6]. Predicting air pollutant concentrations in advance is the basis for enhancing air pollution prevention and achieving comprehensive environmental management, which is important for public health and government decision-making [7].

However, predicting air quality concentrations is difficult, and it is not only susceptible to other factors, such as meteorological factors (temperature, relative humidity, wind speed, and precipitation), traffic pollution, industrial emissions, and so on. It is also affected by the concentration of other pollutants in the air. Specifically, the temperature has an effect on atmospheric and ventilating conditions. Humidity and precipitation have an effect on the deposition of particulate matter, while wind speed contributes to the diffusion of particulate matter [8]. Traffic pollution and industrial emissions will produce some harmful gases such as SO_2_, NO_2_, O_3_, and CO. Some related analyses show that O_3_ will inhibit the growth of PM2.5, PM10, NO_2_, CO, and SO_2_, and PM2.5, PM10, and CO are strongly correlated in each season (correlation coefficient is generally >0.5) [9]. So these effects also pose challenges for air quality predictions. At the same time, air quality prediction is not only spatially dependent. In the time dimension, the PM2.5 concentration of the experimental site is also affected by the air mass concentration of the site in the past, so we need to capture both spatial dependence and time dependence.

In order to solve the above challenges, the author extracted the concentration data of the five sites with the closest correlation with the experimental site and the highest correlation of PM2.5 concentration sequence data to extract its spatial dependence; then the meteorological factors and other pollutant data at the same time were merged as the next input. Next, the previously integrated time-series data with hysteresis *p* were input into the LSTM neural network with *n* hidden layers and one fully connected layer for training. The LSTM neural network is used to extract its time dependence, which will be combined to extract spatiotemporal features and output future predictions of PM2.5. LSTM is a time-cycle neural network that effectively solves long-term dependency problems and avoids gradient disappearance and explosion. It has been proposed to predict future output with past inputs. Compared with the traditional recurrent neural network (RNN) [10], it is unique in that it is designed with a loop body structure that has proven to be very suitable for prediction based on time-series data. And the disappearing gradient problem has better performance than RNN [11–13].

The contribution of this paper mainly includes three aspects: first, this paper proposes a new air quality spatiotemporal prediction model to predict future air quality. Second, integrating historical time air quality data, air quality data from nearest neighbors, meteorological data, and other pollutant data can improve prediction accuracy and help models better predict changes in air quality. Third, the paper evaluates the proposed model on the hourly concentration data set from January 1, 2016, to December 31, 2017, in Beijing. The experiment demonstrates the effectiveness of the method.

The rest of the paper is organized as follows: Section 2 describes the related work; the data and methods used in the experiments are detailed in Section 3; Section 4 reports on the results and discussion; and summary and outlook are drawn in Section 5.

## 2. Related Work

In recent years, more and more researchers use deep learning neural network technology to overcome the problems in the fields of big data and artificial intelligence, which is mainly due to its ability to realize effective learning of feature representation from massive input data and to deeply analyze the potential deep-seated features between data. Because the problem of environmental pollution has become more and more serious in recent years, people pay more attention to health problems, and the relevant environmental health departments have also strengthened management and monitoring, which has led more and more researchers to focus on the research of air quality. Researchers have carefully studied and put forward many prediction models of air quality, which can be roughly divided into three types: single prediction model. It uses the existing methods to predict the air quality data. For the improved prediction model, there are various deficiencies in the prediction of a single model, so the research institute can improve the prediction performance of the existing methods by improving the corresponding weight parameters, adding optimization algorithms, or adding various auxiliary data on the basis of the existing method research: joint prediction model. Although the improved model can make up for the shortcomings of a single model to a certain extent, there are still some limitations. Therefore, researchers continue to make in-depth exploration, combine two or more single models together, give full play to their respective advantages, learn from each other, and combine to further improve the prediction accuracy of the model.

### 2.1. Spatiotemporal Prediction

In recent years, as air pollution has been paid more and more attention, researchers have also proposed many spatiotemporal prediction models to achieve future predictions of air quality. Qi et al. [14] proposed a novel combined prediction scheme based on CNN and LSTM for urban PM2.5 concentration; the model uses CNN to extract the spatial characteristics of inputs between monitoring stations and uses LSTM to predict future air pollution concentrations by learning the characteristics contained in past air pollution concentration time-series data. Zhou et al. [15] proposed a hybrid model for spatiotemporal forecasting of PM2.5 based on graph convolutional neural network and long short-term memory; the model applies a graph convolutional network (GCN) to extract the spatial dependence between different sites and LSTM to capture the time dependence between observations at different times. Wen et al. [16] proposed a deep multioutput LSTM (DM-LSTM) neural network model that was incorporated with three deep learning algorithms (i.e., mini-batch gradient descent, dropout neuron, and L2 regularization) to configure the model for extracting the key factors of complex spatiotemporal relations. Wang and Song [17] proposed a novel spatiotemporal convolutional long short-term neural network for air pollution prediction; high-level spatiotemporal features are extracted by a combination of convolutional neural network (CNN) and long short-term memory neural network (LSTM-NN), and meteorological data and aerosol data are integrated to improve model prediction performance. Box and Jenkins [18] proposed a deep spatiotemporal ensemble model for air quality prediction; the model combines the collection method of the partitioning strategy based on the weather pattern and finds the spatial correlation by analyzing the causal relationship between the sites and generating the spatial data as relative sites and relative regions; finally, the depth LSTM-based time predictor is used to learn the long- and short-term dependence of air quality. These models achieve spatiotemporal prediction by analyzing spatiotemporal data, but the model proposed in this paper is different from the above mentioned. This paper proposes a new air quality spatiotemporal prediction model to integrate experimental air quality data of the site, air quality data of nearest neighbors, meteorological data, and other pollutant data and combine LSTM deep neural network to extract spatiotemporal feature and ultimately achieve future predictions.

### 2.2. Classical Model for Time-Series Prediction

Forecasting flow in a spatiotemporal network can be viewed as a time-series prediction problem. Existing time-series models such as the autoregressive integrated moving average model (ARIMA [19]), seasonal ARIMA [20], and the vector autoregressive model [21] can capture the temporal dependencies very well, yet it fails to handle spatial correlations.

### 2.3. Neural Networks for Sequence Prediction

Neural networks and deep learning [22] have gained numerous successes in the fields such as compute vision [23], speech recognition [24], and natural language understanding [25]. Recurrent neural networks (RNNs) have been used successfully for sequence learning tasks [26]. The incorporation of long short-term memory (LSTM) [27] or gated recurrent unit (GRU) [28] enables RNNs to learn long-term temporal dependency. Some researchers have come up with some bold ideas that combine recursive neural networks with recurrent neural networks to process time-series data, which may better capture the spatiotemporal characteristics of the data. However, as the depth of the network increases, the training cost will also increase greatly, and training will become more and more difficult. Is there any way to improve the accuracy of model prediction without increasing the difficulty of training? It is the direction of future researchers and the problems to be solved.

## 3. Data and Method

### 3.1. Research Areas and Data

**Figure 1:**
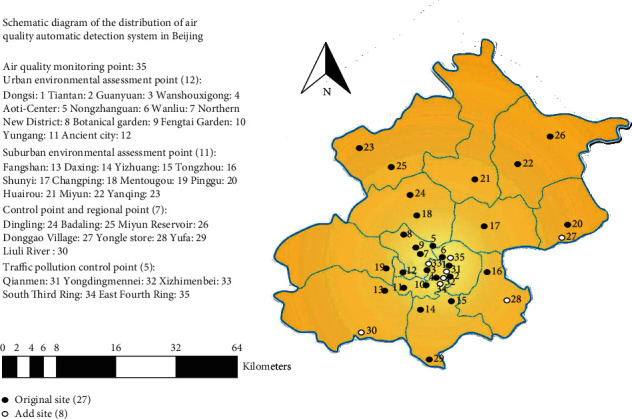
Research area and monitoring site distribution map.

The research area is Beijing, and the data come from the hourly data of 35 air monitoring stations and meteorological monitoring stations in Beijing. The air quality data of Beijing from January 1, 2016, to December 31, 2017, came from the website of Beijing Environmental Protection Testing Center (https://www.bjmemc.com.cn/). The location maps of Beijing and 35 monitoring stations are shown in Figure 1. This paper renumbered the samples and predicted the PM2.5 concentration in representative sites. The S1 station is the urban environmental assessment point; the S17 and S23 stations are the suburban environmental assessment points; the S29 station is the control point and the regional point; and S31 is the traffic pollution monitoring point. Air quality data is collected every hour; there are around 17,000 records for each site. Auxiliary data includes simultaneous meteorological data (temperature, dew point, pressure, wind direction, and wind speed) and other pollutant data (SO_2_, NO_2_, O_3_, and CO), which have also been shown to be highly correlated with PM2.5 concentration [29–33]. The meteorological data come from the National Climate Data Center (NCDC), and other pollutant data are also from the Beijing Environmental Protection Testing Center website. The data sets used in the research are available directly from the website https://beijingair.sinaapp.com/. The data set is first filled with outliers and missing values, normalized, and scaled to [0,1]. The data record of each site is different. This paper selects 67% of the total number of data records as the test set, and the remaining 33% records as the test set.

### 3.2. Extraction for Spatial Factors

According to Tobler's First Law of Geography, everything is related to other things, and similar things are more closely related, that is, the influence of the neighboring sites on the experimental site is greater than that of the distant sites. To illustrate the spatial characteristics of the PM2.5 concentration sequence, the authors calculated the distance between two sites and the Pearson correlation coefficient for the PM2.5 concentration sequence at each of the two sites.

The Haversine formula is used as recommended by Wikipedia to calculate the distance between two sites based on the latitude and longitude of each site. This formula uses a sine function to maintain enough valid numbers even if the distance is small. The formula is as follows:

(1) $$\begin{array}{c} \text{haver}\sin\left(\frac{d}{R}\right)=\text{haver}\sin\left(\varphi_{2}-\varphi_{1}\right)+\cos\left(\varphi_{2}\right)\text{haver}\sin\left(△\lambda \right), \end{array}$$

where

(2) $$\begin{array}{c} \text{haver}\sin\left(\theta \right)=\;\sin^{2}\left(\frac{\theta }{2}\right)+\cos\left(\varphi 2\right)=\frac{\left(1-\cos\left(\theta \right)\right)}{2}, \end{array}$$

where haversin(·) indicates the distance between stations, *R* is the radius of the Earth and can take an average of 6371 km, *φ*_1_ and *φ*_2_ indicate the latitude between two points, and ∆*λ* represents the difference between two latitudes.

The Pearson correlation coefficient is used to measure the linear correlation strength between continuous variables. The formula is as follows:

(3) $$\begin{array}{c} r\left(si,sj\right)=\frac{\text{Cov}\left(s_{i},s_{j}\right)}{\sigma \left(\left(s_{i}\right)\sigma \left(s_{j}\right)\right)}, \end{array}$$

where *r* represents the correlation coefficient of the sequence of PM2.5 concentration between sites, Cov is the covariance, *σ* is the standard deviation.

**Figure 2:**
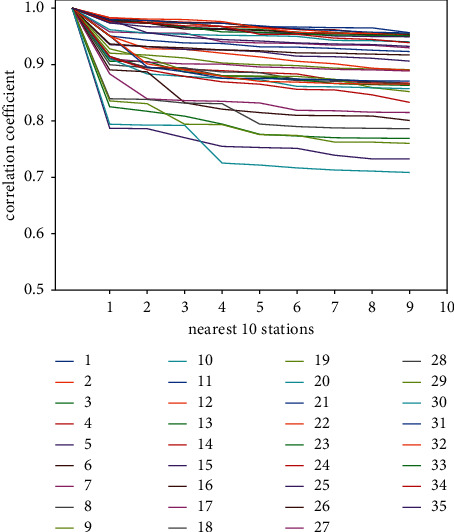
Correlation coefficient of 10 stations with the highest correlation of PM2.5 concentration sequences in 35 stations.

**Figure 3:**
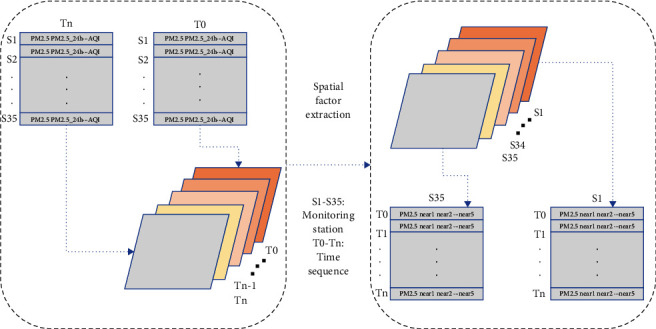
Spatial factor extraction.

The correlation coefficients of the 10 stations with the highest PM2.5 concentration sequence correlation between each of the 35 sites are shown in Figure 2. It can be observed from the figure that the value of the correlation coefficient of most stations is greater than 0.7, so adjacent stations can be used to improve the prediction accuracy of the station. The process of spatial factor extraction is shown in Figure 3. The initial data set used in this paper is the time-series data collected by 35 stations per hour. The data set includes five features, PM2.5 (time average concentration of particles with aerodynamic diameter ≤2.5 mm), PM2.5_24 h (daily average concentration of particles with aerodynamic diameter ≤2.5 mm), PM10 (time average concentration of particles with aerodynamic diameter ≤10 mm), PM10_24 h (daily average concentration of particles with aerodynamic diameter ≤10 mm), and AQI (air quality index), then we will calculate the correlation coefficient between the stations by the formula mentioned above, and extract the concentration of the top 5 stations with the highest correlation with the experimental site, and finally obtain the separate time-series data of 35 stations. Each data record includes six characteristics (self PM2.5 concentration, adjacent station 1_PM2.5 concentration, adjacent station 2_PM2.5 concentration, adjacent station 3_PM2.5 concentration, adjacent station 4_PM2.5 concentration, adjacent station 5_PM2.5 concentration), as the initial data for the next stage for use. As for the temporality of PM2.5 distribution, relevant research has pointed out that the current moment of the station has a good correlation with a certain moment in the past. In order to further reflect the spatiotemporal correlation between the sites, the site timing data obtained above is combined with the auxiliary data including meteorological data and other pollutant data, and the delayed timing values are input into the model. Then long- and short-term memory neural networks are applied to extract their spatiotemporal correlation [34].

### 3.3. ST_LSTM Model

**Figure 4:**
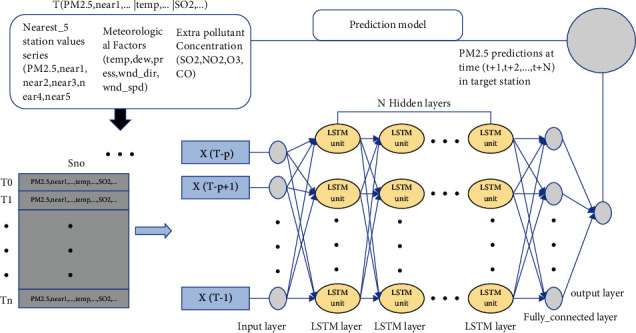
Overall model prediction architecture.

The prediction framework of the model proposed in this paper is shown in Figure 4. The input of the model includes the fusion of three parts of data, including site autocorrelation concentration and adjacent site concentration data, meteorological data (temperature, dew point, pressure, wind direction, and wind speed), and other pollutant data (SO_2_, NO_2_, O_3_, and CO). The output is the predicted value of the experimental site PM2.5 at (*t* + 1, *t* + 2,…, *t* + *N*). The model is divided into three parts: extraction of site autocorrelation concentration and related concentration data of adjacent sites, the fusion of auxiliary data and extraction of spatiotemporal features, and prediction of future PM2.5 concentration.

The first part is the extraction of spatial factors. That is to say, the site self-correlation concentration and the concentration data of the adjacent sites are extracted. The specific content is described in detail in Section 3.2.

The second part is the fusion of auxiliary data. Auxiliary data are added to extract more spatiotemporal features when the model is trained. All data are processed through cleaning and missing values before use, and records with outliers are deleted. For the PM2.5 concentration values, meteorological data, and other pollutant data for each site, the authors used the method of mean filling, fixed value filling, and interpolation filling to process the missing values. The merged data is normalized as an input to the next stage.

The last part is the extraction of spatiotemporal features and the prediction of future PM2.5 concentration. The temporal and spatial features of the normalized time-series data are extracted using an LSTM model with multiple hidden layers. The predicted sequence value at the time of (*t* + 1, *t* + 2,…, *t* + *N*) is predicted using data with a lag of past time *t*.

## 4. Experiments

### 4.1. Evaluation

In order to evaluate the performance of the model proposed in this paper, the author used three evaluation indicators, namely the mean absolute error (MAE), root-mean-square error (RMSE), and the decision coefficient (*R* squared, *R*^2^). Because of the limitations of RMSE and MAE, that is, the same algorithm model, solving different problems cannot reflect the pros and cons of this model for different problems. Because the data is different in different practical applications, it is impossible to directly compare the predicted values, so it is impossible to judge which model is more suitable for predicting which problem. Therefore, the prediction results are converted into accuracy, and the results are all between [0,1]. For the prediction accuracy of different problems, it can be compared and judged which model is more suitable for predicting which problem. *R*^2^ is the best indicator of linear regression. The calculation formula for the three indicators is as follows:

(4) $$\begin{array}{c} \text{MAE}=\frac{1}{m}\sum_{i=1}^{m}\left|\overset{\left(i\right)}{\underset{\text{test}}{y}}-{\hat{y}}_{\text{test}}^{\left(i\right)}\right|, \end{array}$$

(5) $$\begin{array}{c} \text{RMSE}=\sqrt{\frac{1}{m}{\sum_{i=1}^{m}\left(\overset{\left(i\right)}{\underset{\text{test}}{y}}-{\hat{y}}_{\text{test}}^{\left(i\right)}\right)}^{2}}, \end{array}$$

(6) $$\begin{array}{c} R^{2}=1-\frac{\sum_{i=1}^{m}{\left(y_{\text{test}}^{\left(i\right)}-{\hat{y}}_{\text{test}}^{\left(i\right)}\right)}^{2}}{\sum_{i=1}^{m}{\left(y_{\text{test}}^{\left(i\right)}-{\hat{y}}_{\text{test}}^{\left(i\right)}\right)}^{2}}=1-\frac{\text{MSE}\left({\hat{y}}_{\text{test}},y_{\text{tset}}\right)}{\text{Var}\left(y_{\text{test}}\right)}, \end{array}$$

where *y*_test_^(*i*)^ indicates the predicted value and *y*_test_ represents the true value. The smaller the value of MAE and RMSE, the smaller the model error and the better the prediction performance. The larger the value of *R*_2_ the better the model effect, the maximum value is 1; when *R*_2_ is 1, the prediction model does not have any mistakes; when *R*_2_ is 0, the model is equal to the reference model; when *R*_2_ is less than 0, it means that the learned model is not as good as the benchmark model.

### 4.2. Settings

In the prediction architecture proposed in this study, several super parameters are preset, including the number of LSTM layers, the number of neurons in each LSTM layer, the number of fully connected layers, the number of neurons in each fully connected layer, and time step. While fixing other parameters, the impact of each parameter on the prediction performance of the model is checked to determine the best parameters.

Table 1 details the error size of the prediction results obtained using different hidden layers. The data show that when the number of hidden layers is 3, the error is the smallest. Therefore, this study uses an LSTM network with three hidden layers. The number of neurons in each layer is 100; the number of fully connected layers is 1; and the number of neurons in each layer is 1. In addition, other parameter settings in the research are as follows: Adam algorithm is used as the optimization algorithm; Mae function is used as the cost function; the batch size is 128; the tanh function is used as the excitation function of this study; the maximum number of iterations is 100; the learning rate is 0.01; and the performance of the model is the best. In this study, MAE, RMSE, and *R*^2^ are used as indicators to determine the impact of time step on prediction performance. Relevant studies have pointed out that a small time step cannot ensure sufficient long-term memory input of the model, but a large time step allows too many irrelevant inputs to be added [50].  Table 2 shows the impact of different time steps on prediction performance. It can be observed from the table that when the time step is 14, the performance of the model is the best.

### 4.3. Baselines

In order to test the overall performance of the current model, the author conducted a series of comparative experiments on two types of baseline models: nondeep learning model and deep learning model.

1. Nondeep learning baseline model. This includes linear regression (LR) models, support vector regression (SVR) models, random forest (RF), and autoregressive moving average (ARMA) models.
2. Deep learning baseline model. That is, it contains the different components in the model presented in this paper. It includes LSTM_N model (with only spatial factor data), LSTM_NW model (with spatial and meteorological data), LSTM_NE model (with spatial and other pollutant data), LSTM_WE model (with meteorological and other pollutant data), LSTM_S model (without any auxiliary data), and ST_LSTM model (model presented in this paper).

## 5. Results and Discussion

### 5.1. Prediction Performance

**Figure 5:**
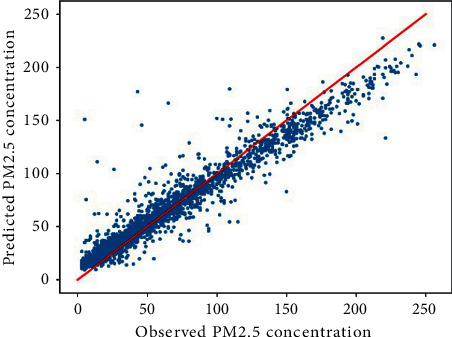
Scatter plot of PM2.5 observations and predicted values.

After determining the optimal network architecture for the current prediction task, the training set is used to train the current ST_LSTM model until convergence and then evaluated on the test set. This paper predicts the PM2.5 concentration value for the next hour for the monitoring station numbered 1 in Beijing and compares the predicted value of the model with the real value. The Beijing No. 1 site drawn using the ST-LSTM model proposed in this paper predicts the PM2.5 concentration value and the observed PM2.5 concentration value for the next hour as shown in Figure 5. It can be observed from the figure that the predicted value is substantially consistent with the observed value. The *R*^2^ value between the observed and predicted data indicates that the model can capture 93% of the interpreted variance. The feasibility and accuracy of the proposed model are verified.

**Figure 6:**
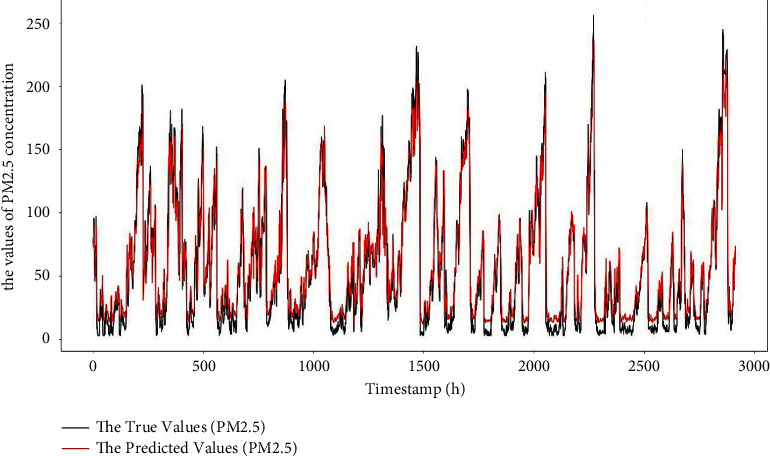
Fit curve of PM2.5 real value and predicted value.

At the same time, the author also plotted the curve of the true value and the predicted value on the test set. The plot of the true value and the predicted value of the test site of Beijing No. 1 is shown in Figure 6. From the figure, the author observes that the trend of the two curves is roughly the same, and the degree of fitting is better. It is indicated that the model proposed in this paper can accurately capture the temporal and spatial variation of PM2.5 and achieve a relatively accurate prediction of air quality in the future (predicted future concentration of PM2.5).

### 5.2. Comparison of Experiments

The comparison of the predicted performance of the model proposed in this paper with the other eight baselines at the next hour is shown in Table 3 on the three evaluation indicators of MAE, RMSE, and *R*^2^. It can be found that the deep learning baseline model performs better than the nondeep learning baseline model, where the SVM nondeep learning baseline model performs the worst. Comparing the three evaluation indicators of all deep learning baseline models, it is found that the proposed model performs optimally in predicting performance. Due to data limitations, the authors only conducted a comparison of the predicted results of the next 1 to 6 hours of the deep learning baseline model. The numerical values of the above six deep learning models on the MAE indicator are shown in Table 4 and are used to predict the air quality from the 1st hour to the 6th hour. From the numerical values, we can observe the predictions in the next 1 to 3 hours and the 6th hour. The error of the model proposed in this paper is the smallest. The error of the LSTM_NE model is the smallest at the fourth and fifth moments. The performance of the prediction model proposed in this paper ranks second; the possible reason is that other pollutant factors have a greater impact on PM2.5 concentration. The LSTM_NE model only considers other contaminant factors, and the model proposed in this paper not only considers other pollutant factors but also considers meteorological factors. Considering more factors, it weakens the influence of other pollutant factors. From the comparison of the data of the two models LSTM_NW and LSTM_NE, the author observed that the prediction performance of the second to sixth moments except for the 1st hour, the error of the LSTM_NE model is smaller than the error of the LSTM_NW model. It also echoes the above conjecture that the influence of other pollutant factors on PM2.5 concentration is greater than that of meteorological factors on PM2.5 concentration.

## 6. Conclusions and Outlook

This paper presents a spatiotemporal prediction model for future prediction of air quality based on long short-term memory (LSTM) neural network. The model achieves more accurate and stable future predictions by integrating historical time air quality data, air quality data from nearest neighbors, meteorological data, and other pollutant data into the model. At the same time, the author used the real data sets of Beijing from January 1, 2016, to December 31, 2017, to evaluate the model proposed in this paper using MAE, RMSE, and *R*^2^ evaluation indicators. The validity of the model is presented in the paper.

Generally, the proposed model is suitable for processing data from multiple monitoring sites in a single city as an input to a time series that can combine the interaction of multiple sites with the time dependence of air pollutants in the prediction system. However, there are still some limitations: (1) it can only predict the air pollutant concentration of a single site in a single city and cannot achieve the overall forecast of the city. In the future, it is hoped that all the site data and site prediction data in the city can be combined to achieve a comprehensive forecast for the entire city. (2) The model proposed in the article is only evaluated on the Beijing data set and has certain limitations. In the future, it is hoped that more monitoring data of other urban monitoring sites can be collected to further verify the performance of the model. (3) In the future work, I hope to consider more impact factors, such as traffic flow. This will allow you to better capture changes in air quality and obtain more accurate predictions.

## Figures and Tables

**Table 1:** Comparison of different hidden layers on model performance.

Hidden layers	MAE (*μ*g/m^3^)	RMSE (*μ*g/m^3^)	*R* ^2^

1	7.57	12.261	0.944
2	7.54	13.250	0.944
3	7.34	11.963	0.947
4	7.99	12.260	0.944
5	7.69	12.407	0.943

**Table 2:** Comparison of model performance with different time lags.

Parameter	Parameter (*h*)	MAE (*μ*g/m^3^)	RMSE (*μ*g/m^3^)	*R* ^2^

Time lag	1	7.81	12.727	0.92
	2	8.26	13.020	0.92
	4	7.31	12.374	0.92
	8	7.26	12.219	0.93
	10	7.06	12.132	0.93
	12	7.03	12.074	0.93
	**14**	**6.83**	**12.048**	**0.93**
	16	6.99	12.082	0.93
	24	7.33	12.393	0.92

**Table 3:** Comparison of various baseline models with the model's indicators for the next hour.

Baseline model	MAE (*μ*g/m^3^)	RMES (*μ*g/m^3^)	*R* ^2^

LR	13.08	23.707	0.903
RF	8.02	15.131	0.918
SVM	34.69	68.106	0.255
ARMA	7.46	13.958	0.923
LSTM_N	7.11	13.943	0.922
LSTM_NW	7.33	13.567	0.926
LSTM_NE	7.79	12.757	0.919
LSTM_WE	7.21	12.353	0.926
LSTM_S	7.81	14.664	0.916
ST_LSTM	**6.81**	**12.080**	**0.930**

**Table 4:** Mean absolute error (MAE) values for different models predicted for the 1st to 6th hour of each baseline.

MAE (*μ*g/m^3^)	1st hour	2nd hour	3rd hour	4th hour	5th hour	6th hour

LSTM_N	7.02	10.78	14.09	17.27	20.25	22.40
LSTM_NW	7.33	11.02	14.03	16.71	19.15	21.19
LSTM_NE	7.79	10.33	13.24	**15.54**	**17.88**	20.26
LSTM_WE	6.99	10.37	13.18	16.98	18.09	19.85
LSTM_S	7.81	12.19	14.59	17.95	19.81	22.56
ST_LSTM	**6.81**	**10.22**	**12.87**	15.92	18.53	**19.69**

## Data Availability

The data used and analyzed in this paper are available at https://www.bjmemc.com.cn/.
